# Best practice in bereavement photography after perinatal death: qualitative analysis with 104 parents

**DOI:** 10.1186/2050-7283-2-15

**Published:** 2014-06-23

**Authors:** Cybele Blood, Joanne Cacciatore

**Affiliations:** School of Social Work, Arizona State University, Phoenix, USA

**Keywords:** Perinatal death, Stillbirth, Neonatal, Parental grief, Bereavement photography, Psychosocial interventions

## Abstract

**Background:**

Postmortem memento photography has emerged in Western hospitals as part of compassionate bereavement care for parents facing perinatal death. Many parents endorse this psychosocial intervention, yet implementation varies greatly and little research on parents’ specific needs guides health care professionals. Parents are in crisis and vulnerable after the death of their child, thus best practice is crucial. This study contributes 104 parents’ experiences and opinions toward the understanding of best practice in perinatal bereavement photography.

**Methods:**

Parents who experienced the perinatal death of their child were recruited from U.S.-based bereavement organizations and social media sites. Volunteers completed an anonymous internet survey with open- and closed-ended questions. Direct recommendations and pertinent statements regarding the process of postmortem photography were analyzed for thematic content in keeping with conventional content analysis. Recurrent themes and sub-themes were counted to identify response patterns.

**Results:**

Of 93 parents with pictures, 92 endorsed them. Of 11 without pictures, nine wanted them. Parents made a variety of recommendations regarding appropriate psychosocial support, the consent process, obstacles to photography, logistics of photography, and material aspects of photographs themselves. Overall, parents wanted many pictures and much variety. Some wanted professional photography while others wanted support for taking their own pictures. Parents wanted guidance from staff who respected their particular needs. Many said decisions were difficult during their crisis. Parents who were initially resistant expressed current appreciation for pictures or expressed regret that they had not participated. Parents recommended that professionals strongly encourage parents to create memento photos despite parents’ initial reservations. Persistent cultural reasons against photography emerged in one case. Quotes by parents illuminate themes and enable respondents to speak directly to health care professionals.

**Conclusions:**

Parents overwhelmingly support postmortem bereavement photography when conducted sensitively, even if imperfectly executed. Providers significantly influence parents during their crises; mindful, patient-centered care with appropriate respect for difference is necessary. Providers must understand the importance of postmortem photographs to parents who have limited opportunity to capture memories of their child. Hospitals should provide education and support for this important psychosocial intervention.

**Electronic supplementary material:**

The online version of this article (doi:10.1186/2050-7283-2-15) contains supplementary material, which is available to authorized users.

## Background

Psychosocial hospital care after perinatal death has changed significantly in recent decades throughout the United States and Europe. Until the 1970s, medical staff often explicitly prevented parents from seeing and holding their stillborn babies and encouraged families to quickly forget their loss (Erlandsson et al. 2013; Lovell 1997; Stringham et al. 1982). Research then began to identify adverse psychological outcomes attributable to widespread disregard for the needs of parents facing perinatal death (Stringham et al. 1982; Lewis and Page 1978; Malacrida 1999). Grief theory acknowledged *continuing bonds* (Klass et al. 1996) as healthy and facilitative in the mourning process of many bereaved parents (Davies 2004; Klass 2006). Parental organizations advocated for paradigm change (Lovell 1997; Malacrida 1999), and revised understandings of appropriate psychosocial support for these parents led many facilities to adopt more compassionate perinatal death protocols (Gold et al. 2007; Lang et al. 2011).

The nature and quality of perinatal bereavement services vary widely, however (Cacciatore and Bushfield 2007; Harvey et al. 2008; Kelley and Trinidad 2012). Research still finds perinatal death marginalized and minimized in medical environments (Lang et al. 2011; Cacciatore and Bushfield 2008; Frøen et al. 2011). Some families face provider insensitivity when their cultural or personal preferences conflict with standardized protocols (Chichester 2005; Hughes and Goodall 2013; Kendall and Guo 2008). Parents continue to report emotional, spiritual, or practical needs unfulfilled in hospitals, which increases family distress during and after a newborn’s death (Lang et al. 2011; Cacciatore and Bushfield 2007; Einaudi et al. 2010).

Grieving parents appreciate when health care professionals convey respect for their particular needs and acknowledge the significance of their loss (Cacciatore 2012; Henley and Schott 2008). They value empathetic, humble, consistent, and honest communication during their crisis (Kelley and Trinidad 2012; Cacciatore 2011; Williams et al. 2008). Most cherish mementos such as plaster cast footprints, locks of hair, infant clothing worn in the hospital, and photographs of their baby, all of which may be collected by hospital staff as part of bereavement caregiving (Kelley and Trinidad 2012; Williams et al. 2008; Kavanaugh and Moro 2006).

### Photography in perinatal bereavement care

Researchers and parents agree that perinatal bereavement interventions should validate the baby’s worth and existence, support parents’ expression of grief and mourning, assist in meaning-making, and improve parents’ ability to cope with the death (Murray et al. 2000; Blood and Cacciatore 2014; Wheeler 2001; Capitulo 2005). Research has elucidated the value of bereavement photographs for these tasks (Blood and Cacciatore 2014; Capitulo 2005; Riches and Dawson 1998), yet often in perinatal death the only opportunities to capture the baby’s visage occur postmortem. A vast majority of U.S. and European parents participating in research report gratitude for postmortem photographs of their newborn; conversely most express regret if they do not have such photos (Gold et al. 2007; Harvey et al. 2008; Blood and Cacciatore 2014). Two studies have identified postmortem bereavement photography as “one of the most helpful services” during the crisis of a newborn’s death [(Gold et al. 2007), p. 1160].

Photographing the dead, especially children, was widely practiced from the advent of photography until the early 20^th^ century (Hilliker 2006; Burns 2002). As death became an increasingly institutionalized and socially taboo matter, postmortem photography disappeared from mainstream American culture, though it remained acceptable and even popular among some immigrant groups (Hilliker 2006; Burns 2002). Since the 1980s it has made a strong mainstream comeback, as providers incorporated the wishes of many bereaved U.S. and European parents to have material acknowledgement and mementos of their stillborn children (Malacrida 1999; Layne 2000).

Though a wide variety of cultures past and present have embraced postmortem photos as an aid to memory, mourning, narrative, and therapeutic grief ritual, some in the general public—and in health care—still perceive these practices as strange or morbid (Hilliker 2006; Burns 2002; Cacciatiore and Flint 2012; Johnson 1999; Kavanaugh and Hershberger 2005; Jones 2002). Additionally, some cultural traditions do not endorse memento-making practices. “Parents from some cultures or religious traditions might not want or be permitted to take photographs of their dead children. Some Native American tribes, The Church of the Latter-Day Saints, Old Order Amish, and Orthodox Jewish traditions have proscriptions against postmortem photography, contact with dead bodies, or both” [(Blood and Cacciatore 2014), p. 226; (Chichester 2005; Stamm and Stamm 1999)]. Muslim families, also, may not want postmortem photography or memory boxes (Lundquist et al. 2003; Hébert 1998). Cultural sensitivity by providers is an imperative, remembering that “cultural groups are not homogeneous, and individual variation must always be considered in situations of death, grief, and bereavement.” [(Clements et al. 2003), p. 19].

### Implementing bereavement photography services

For the majority of parents who do wish for concrete memories of their infant, the enactment of postmortem bereavement photography remains unfortunately inconsistent (Gold et al. 2007; Harvey et al. 2008). Flexible protocol has been proposed by professional associations and nonprofit organizations (Gold et al. 2007), and professional articles have advised technical and practical details [e.g. (Jones 2002; McCartney 2007; Meredith 2000)]. Yet, some hospitals neither mention nor offer postmortem photography to parents, and those that do vary greatly in their means of implementation. Some facilities call on professional photographers, while others assign nurses to take photographs or simply offer parents a disposable or digital camera (McCartney 2007).

Parents facing the intense crisis of child death are vulnerable to the attitudes and intimations of healthcare providers (Cacciatore 2011; Badenhorst and Hughes 2007; Limbo and Kobler 2010). Providers should not allow personal opinion about postmortem photography to guide their approach to parents, but instead should advise according to best practice evidence and the insight gained from attending mindfully to individual parents’ feelings, wishes, and needs (Hughes and Goodall 2013; Cacciatore 2012; Limbo and Kobler 2010). To date, academic literature contains scant mention of specific parental preferences for the enactment of postmortem photography (Harvey et al. 2008). Studies either encompass multiple aspects of bereavement care, only superficially addressing photography, or rely on very small samples [see (Gold et al. 2007; Harvey et al. 2008)]. The present study on the needs and desires of parents in regard to postmortem photography after perinatal death was conducted with data from 104 bereaved parents. Verbatim presentation of indicative data will illuminate parental recommendations and further the understanding of best practice in the field of perinatal bereavement care.

## Methods

### Participants and procedures

The present study data were obtained as part of a broader research project with 181 parents whose children died at any age. Following approval by the Arizona State University Institutional Review Board, respondents were recruited with online advertisements and invitation emails targeting U.S.-based bereavement organizations. Invitation was clearly extended both to parents who did and did not have experience with postmortem photography. From late October 2011 until April 2012 participating parents completed a 15-minute, anonymous online survey with closed- and open-ended questions and ample opportunity for narrative construction [see Additional file 1]. Quantitative and qualitative exploration of these data, including analysis of the meaning of photography to parents, has been presented elsewhere (Blood and Cacciatore 2014).

### Data reduction and analysis

The present study defined perinatal death as combined stillbirth and neonatal death (20 weeks gestation to 28 days after live birth). This definition is used by the U.S. National Center for Health Statistics and suggested in *Pediatrics* journal (Barfield and Committee on Fetus and Newborn 2011). Analyses were conducted with data from parents whose children died perinatally (*n* = 132). Twenty respondents provided no answers to open-ended questions, thus their responses contained no recommendations and were removed. Eight referred solely to the value they accord their pictures, offering no comment on procedure or recommendations. Statements indicating the high value of postmortem photography were ubiquitous in the broader data (Blood and Cacciatore 2014); as this has been discussed elsewhere these eight responses were also removed. Thus, final data for this study included 104 participants.

In accordance with conventional content analysis techniques (Hsieh and Shannon 2005) in the context of qualitative descriptive methodology (Sandelowski 2000; Sandelowski 2010), data were inductively analyzed for responses that could specifically inform photography practice. Useful responses comprised two categories: 1) overt recommendations phrased in the third person or overt statements wishing procedures to have been different during the experience (e.g., “I so wish someone had offered professional photos”), and 2) narrative or commentary on procedure that enhances understanding of parents’ needs (e.g., “I was reluctant, but the perinatal counselor suggested it and I am so thankful I listened”). Further analysis accreted specific statements with similar or closely related content into themes. Each theme was counted only once per respondent regardless of repetition to quantify how many parents addressed each topic.

## Results

### Descriptive statistics

Of the final 104 parents in the current study, 93 (89.4%) possessed postmortem photos taken for bereavement or memorial purposes, and 11 (10.6%) did not. Of the 104 deceased children represented in this study, 45.6% died in 2010 or later and 74.8% in 2006 or later. 9.7% of children died prior to 2000, with two in the 1980s, seven in the 1990s, and one outlier in 1958.

Of 102 parents who reported their sex, 101 were female. Of 101 parents who reported their ethnicity, 86 (85.15%) identified as Caucasian or White, 3 (2.97%) as African American or Black, 5 (4.95%) as Latino/Hispanic; 3 (2.97%) as Native American/American Indian, and 4 (3.96%) as Asian American, Asian, or Pacific Islander. The age of respondents ranged from 21 to 74, with a median of 35.5 years, and 80% of parents were 29 to 44 years old (*n* = 102).

### Qualitative analysis

Of the 93 parents with postmortem photographs, 92 expressed overall approval and varying degrees of positive thoughts or feelings about them. Of the 11 parents without photographs, nine expressed some degree of desire for them. Statements by all 104 parents can be summarized in several broad categories. Parents discussed obstacles to photography, photography-related psychosocial and logistical support needs, and details that may have improved the actual photographs. Some comments addressed more than one category.

#### Obstacles to postmortem photography

The most frequently stated factor affecting photography was the parent’s state of crisis, with 31 parents mentioning physical or emotional shock, including being unconscious, “drugged”, or, more frequently, simply unable to keep up psychologically with rapidly unfolding events: “My mind hadn’t quite processed it”. Parents were dissociated, “confused”, “in a fog”, and “numb”. Several mentioned disrupted memory process: “…In such as state of shock that I would not remember what my daughter looked like if it wasn’t for those pictures”.

Twenty-six respondents said the idea of memorial photography did not occur to them during their crisis. “When you have just lost your child you are not thinking clearly. I would have never thought of asking for photos”. “I wouldn’t have thought about having pictures taken until it was offered”. Eighteen parents were initially resistant to the idea or process of postmortem photography but in retrospect strongly endorsed having photographs. “I thought they were out of their mind when the question was raised….In the end I was grateful”. “At first I was annoyed with the nurse….Now that I have the photographs, I am so glad”. “Originally in my grief I didn’t want pictures taken…three years later I realize that any kind of photograph would be an invaluable treasure”. As one said, “The initial thought that pictures are only taken on happy occasions is not the case”.

Several parents said decisions were affected by their psychological condition: “I couldn’t think for myself at the time”. An insightful respondent explained further, “In that state of shock and drowning grief making decisions is really hard, and so it is easier to say no to everything than to have to think about it…[parents] don’t know what to do”. Several said their spouse made the decision for memorial photography when they were incapacitated; others said family members’ opinions made the difference. “My husband signed the paperwork, and boy am I glad he did”. “My mom convinced me…I am very thankful”. “When my sister suggested taking pictures…I thought she was crazy! However, I would be so upset with myself if we did not have pictures of my [baby]”.

Three parents who were in severe medical crisis post-birth emphasized their total reliance on others to create adequate photographs: “I have none of the pictures you would want if you could never see your child again. I wish the nurses, with me in a coma, would have taken more time with her. I was completely crushed”. “These pictures that were taken professional [sic] by the hospital are the only memory I have left of my son. I was on life support until three days after he was delivered”. “I went from being pregnant and then woke up 21 days later from a coma….They took photos with clothes on and bears…I’m so glad the nurses took these”.

In some cases respondents’ families participated, encouraged, or took photos, but two said family opposed photographs: “They thought it was morbid”. “In the beginning my entire family was against us having a photographer present”. Several other obstacles to photography were mentioned by parents. Many raised the issue of camera availability. “I would have never in a million years thought to bring a camera to take pictures of my dead daughter”. Two parents wished to hire a professional photographer, but could not afford it. One of these misunderstood the free volunteer bereavement photography service: “The hospital had a flyer, but there was no information on the price”.

Six parents reported inadequate or rushed time with their child as another obstacle to photography and a broader concern in general. “We felt robbed of our time with our son…the hospital staff made it seem very limited….Many more pictures could have been taken if we had been afforded an opportunity”. “Unfortunately, the hospital didn’t give us much time to spend with him after he died. Hospitals…should give parents as much time as they need”.

#### Supporting parents’ needs

##### Obtaining consent

While ethical practice clearly requires informed consent for non-medical, memorial postmortem photography, 35 parents explicitly endorsed providers strongly recommending and encouraging parents to take photographs. Twenty parents recommended that providers be assertive in educating parents who are initially resistant, or were glad this occurred in their case. “I’m grateful that the NICU nurses and social workers helped me be ok with this idea”. “Grateful that they knew what I would need long before I knew”. “Really encourage families to have pictures taken”. Five urged providers to explain how important most parents find pictures later. Thirteen parents specifically recommended that “even if [they] don’t want them in the moment,” parents should be encouraged to have pictures taken that professionals can hold or store or because they will likely “change their minds later in their grief process”. “It will become very important”. Many parents indicated that the way the idea of photographs is presented matters: “The way the counselor presented it to me worked”. Some specifically said providers should ask multiple times, offering to take and store the photos for parents. One mother reported that being asked again after two hours was the factor that led to her consent. Multiple responses conveyed the importance of normalizing photographs of a baby who died. “I wish so much that someone had told me it was ok to take her picture”. "I didn't realize it was normal for people to take pictures of their dead children”.

##### Supporting both professional and family photography

Twelve respondents suggested providers support and encourage families to take their own pictures. Three suggested help for choosing shots and poses: “To have some kind of guidance, whether it be from another person, [or] photo checklist of possible poses…would be so valuable and appreciated”. Three parents who had professional pictures taken said they wish someone suggested they also take their own, two noting that the baby’s appearance deteriorated by the time a professional photographer arrived. “I wish we had the option of taking photos right after birth, when he was pink and warm”. Five parents said they were glad they took their own pictures. “It made the process an intimate family moment”. Two parents noted that “it is easier now with mobile phones”. Several more parents said the quality of the hospital photos was either compromised or that the shots did not include family. “I regret not taking a photo of us together”. “The five pictures the hospital staff took…don’t look very good and are grainy. I wish [my husband] had been there to take more pictures himself. I’d like health service professionals [to] strongly suggest to parents that they have a digital camera to take pictures”. Eight parents specifically urged providers to offer parents use of a camera. One noted: “[Disposable camera pictures] came out so much better than our cell phone pictures did”. One parent also suggested making a tripod available. Ten parents were glad the hospital offered to take photographs. “To leave the photo taking to the parents is overwhelming…they are already dealing with so much”. “We were in no shape emotionally”. Several suggested, however, that the person taking pictures should undergo training.

Eight parents wished “someone had offered professional photos”, and many parents reported positive experiences with such professionals. Four pointed out that parents would not know about free memorial photography services, and seven suggested providers educate parents about this service, if available. Two suggested a policy to call a professional no matter what parents initially say about the matter because “they will change their mind”.

##### Professionals during the photography process

Nine parents commented favorably on staff’s or their photographer’s manner, noting “kindness”, “thoughtfulness”, unobtrusiveness, and a welcoming attitude. “[Our nurse] showed us, through her openness, that…it was ok to do whatever I wanted”. “The photographer…was so sweet…handled him very lovingly and carefully”. One, however, noted a nurse’s discomfort in taking a photograph, and another noted their photographer’s inability to suggest creative poses. One parent recommended: “It is important for health professionals not to project their own prejudices on parents, and to allow pictures at any stage, no matter how strange this may seem to them”. Another parent’s experience highlights the need for consistent communication by team members: “Had I been asked the actual question as it was asked [of the nurse], I would have understood my options for photography”.

#### Creating quality mementos

Parents notice the quality of pictures. Seven parents mentioned blurred focus or poor lighting; three mentioned the importance of natural light. Two parents appreciated the “tasteful” nature of photos taken. Five parents indicated, though, that any kind of picture was valuable regardless of quality. “They are not great photos, but I so, so [sic] very grateful to have them”. “It means the world to me that we have these photos, as blurry or unsatisfactory as they may be”.

Seven parents noted that some parents may want photographs despite the baby’s appearance. “I am thankful to have something rather than nothing. Even if they are not pretty”. “She was small and physically damaged…I chose not to take pictures. I regret it so much”. “Although it’s evident my son was stillborn and effects of that show in the photos, he is still beautiful, and [he] still matters”. Several parents mentioned retouching as a means to address discoloration and several suggested thoughtful posing: “We chose to take pictures from a distance and with us holding her”, and “When I look at them I will see a child that does not have birth defects but rather a child who is sleeping”.

Seven parents recommended time as a factor: Taking postmortem photographs as soon as possible can improve outcomes. Two parents suggested pictures be taken when the baby is alive, if possible; one parent expressed the wish for such pictures. Two parents urged providers to broach the subject of memorial photographs before the birth or death, so parents might prepare. One also suggested, “Prepare parents for the changes a deceased baby will go through”.

##### Variety and family participation in pictures

Thirteen parents expressed a wish to have more pictures. Eleven without such dissatisfaction had more than one person (or camera) taking photographs. Five respondents reminded parents and professionals to “take lots”, noting that “One can never have too many photos”. Seven parents suggested as much variety as possible; four mentioned photographs of specific bodily detail. “Don't only take regular shots of baby and baby with family (if they choose that) but also of hands and feet especially if it was an early loss…for many parents this allows them to share their baby when otherwise they may not”. Three parents wished they had more detail in their photos. “I didn’t have any photos of [him] after he died without that hat on…or the back of his body…or of his knees…or of him without all the blankets swaddling him”. “The one thing that I did not think was to take pictures of her fingers/hands and toes/feet…especially her little feet as they were in such perfect condition”.

Being involved in the photography process was important to many parents. Fourteen parents emphasized the importance of photos with their baby being held: “I wish I had more of just me and him, me holding him”. “It was very important because we have pictures of US holding our son”. “My only wish is that my husband would've been holding [him] in a few of the photos”. Eight said they valued getting to choose clothing, poses, or photography options for their newborn. “It meant a lot to us to be able to pick the outfit out, his hat, and blanket”. “I remember telling the nurse to take a picture of his ‘boy parts’ and his head so I could remember his hair”. Nine parents wished they had been involved in such decisions during the process. “I would have suggested taking many more photos of all angles, and all parts of his body”. Several parents of neonates who died commented that postmortem pictures gave them a chance to hold their infant without medical apparatus. “My husband and I took photos holding him without any tubes or wires, something we never did while he was alive”.

#### After the pictures are taken

Three parents recommended digitally stored photos in addition to prints. Two recommended pictures be offered in a sealed envelope, and five suggested that, when parents choose not to receive them, hospitals hold the photographs in case they change their minds. One parent specifically appreciated that the volunteer photographer sent a preparatory card before sending the actual pictures. Two parents were surprised by the inclusion of photographs in a box sent home with them by the hospital.

Picture deletion created distress for two parents. One “had problems with a non-bereavement based company…they deleted some of the pics and ‘only gave us the best’ like they do with living children, who people have time to make more memories with!! I was livid”. The other parent had a hospital photographer:

“We only have four photographs and I really wish we had more. When we asked for ALL of the pictures on [digital] media, she said that they had already been deleted. Why was that her decision? That is all we have of our son! To say that we're livid about this is an understatement. Parents should be given ALL pictures taken of their child. Let the parents decide what to do with them”.

#### Parents who were not asked if they wanted photos

Only 82 (78.8%) parents said they were asked if they wanted postmortem memorial photography. Of the other 22 parents who were not asked (21.2%), nine did not have pictures and 13 did. For the 13 families with pictures, five said they were not asked but without any prompting chose to take their own pictures. Of the remaining eight parents, six mentioned no specific concerns about the issue of consent. It is possible a spouse or paperwork signed by these parents gave consent. All six generally approved of having pictures at the time of their study participation, with four very grateful. The seventh parent had given consent for nurses to take photographs but was angry that a family member took an additional photo without permission. “I would like health professionals to please ask before allowing anyone to take photos”.

The eighth parent, however, was deeply disturbed that pictures were taken by nurses without her consent. The pictures and the manner in which the hospital handled her child’s body was a cultural violation to Native American tradition. “I understood it was meant in a good way…but I viewed it as culturally insensitive”. She added, “I was told by the people (Native American advisers) who helped me with my daughter's burial that I should not have photos or keep any item that touched my daughter after her death”, and “I have always felt conflicted about keeping them”.

#### Parents who do not have pictures

Eleven parents did not possess postmortem photographs nor had they been asked if they wanted them. Nine of these 11 expressed discontent with this outcome. One wanted the option, however because she was 16 at the time, she could not contest a decision made by social services against pictures. One parent threw away pictures taken without her assent which were placed in a hospital memory box: “The pictures did not look like the way we remembered him…[they] made him look cold and alone”. However, she now regrets the decision, as “that picture engraved in my mind of exactly how he was…is fading”. She wishes she had pictures “taken with the three of us [with husband]”.

One parent whose baby had a genetic deformity was urged *not* to have pictures, which has led to ambiguous feelings and ongoing, regretful curiosity: “In retrospect, I find myself thinking a lot about how she would have looked. It's possible that I would have regretted going the other way, but I don't know for sure, and that is hard”. Providers, she said, “shouldn't assume anything…and should suggest all options, if they are medically reasonable”.

Three parents of neonates who died had only a small number of pictures from the short time their child was alive and expressed the desire for more, including postmortem. “I would have liked someone to offer me the choice of spending time taking photos with my deceased child”, and “I just wish I knew it was possible”. Two other parents of neonates who died did not want postmortem photography for their infants. One said “I had three weeks with [her]. I took plenty of my own pictures”. Another said “I had taken photos when she was one day old, I wouldn't like to have her photographed dead. I prefer remembering her alive”.

#### Broad appreciation expressed by parents

Parents noticed and appreciated emotional support during their acute grief. “I am so grateful that I delivered at a hospital with a very progressive perinatal loss program”. Fifteen expressed appreciation for health care professionals, photographers, and volunteers who brought compassion, empathy, and thoughtfulness to bereavement photography. “The photographer in the area couldn't make it…I am so glad [the nurse] went the extra mile and did that for us”. “Thankful that a photographer dropped everything on a Saturday evening to come out and help our family”. “[The bereavement photography organization] and the hospital staff went out of their way to make incredible photos that we will cherish for a lifetime”.

## Discussion

For the vast majority of parents in the present study, both the process and product of postmortem bereavement photography were tremendously valuable. Comments both indirectly and directly attesting to the meaning of the photographs were ubiquitous. One parent summarized: “It is critical that professionals understand the importance of photographs”. Numerous comments encompassed the core urgency realized in hindsight: Though parents facing perinatal death are in the midst of overwhelming crisis, the opportunity to create memories is transitory and the decision not to take photographs—or enough photographs—is irremediable. Most without pictures will later feel regret.

A prominent theme in these present data and extant literature is that parents with photos commonly wish they had more (Gold et al. 2007; Blood and Cacciatore 2014). A combination of professionally taken pictures, pictures taken by nursing staff, and self-taken pictures may best ensure maximum quantity, quality, and variety. Many parents wanted a professional photographer. Several dozen parents highly recommended a volunteer organization which engages professional photographers, suggesting providers contact them when no hospital photographer can assist. Present data also indicate that most parents value the opportunity to hold their baby and create memories by participating in the photography session, and many wish they had more choice in the specific angles and poses of photographs.

One third of parents in the present study reported some degree of cognitive impairment after the death of their child, and for many incapacitation was an obstacle to participation in the photography. Several mothers who were unconscious or heavily medicated after birth noted the critical impact of proactive efforts by professionals (or the lack thereof) in capturing their child’s appearance at birth. Many parents had difficulty considering the option of postmortem photography when suggested by clinical staff. Due to the highly traumatic nature of perinatal death, parental decision-making processes are often challenged (Hughes and Goodall 2013); mothers experiencing the death of a baby are vulnerable and may not understand how a decision they make in a moment of crisis may affect them in one year, ten years, or twenty years (Cacciatore 2011).

Indeed, present data suggest that parents initially refusing pictures may wish for them later. One fifth of parents (*n* = 21) described some sort of negative initial response to the proposal, process, or product of postmortem photography, with *all but one* parent later wishing for or appreciative of the pictures. In addition to parental impairment due to trauma, such initial reactions may have reflected social stigma surrounding death, dying, and material representations of mortality, or “personal and cultural beliefs that photography is meant for happy occasions” [(Michelson et al. 2013), p. 515]. Several respondents in the present study felt that time spent by providers educating parents about grief and the value of such photographs would help them make a wise and well-informed decision. Osborne (Osborne 2000) suggests that another hospital team member can re-introduce consent with parents later if they initially say no. In the event that parents are confused or undecided about bereavement photography, it may be appropriate to approach them again and further explain the rationale, citing research and clinical wisdom in a gentle and non-coercive way. Still, a minority of parents, due to culture or personal preference, may refuse pictures, and their decision must be honored. In the current data, one heritage-consistent Native American parent felt photos were a violation, but two other Native American parents, one of whom reported her religion as “indigenous”, endorsed photography. All twelve parents reporting ethnicities other than White or Native American also endorsed the photography.

Two parents in this study reported harm from professional caregivers sending memento pictures home without informed consent. Significantly, no parent of 104 reported being hurt or feeling unduly pressured by professionals encouraging pictures. Though not primed nor prompted by survey questions to specifically discuss the issue of consent, permission, or provider influence on participation in bereavement photography, one-third of respondents overtly endorsed providers’ actions guiding or assertively encouraging them toward such photography. Many parents cited that without this encouragement, they would have lost a crucial opportunity to create mementos of their child. Parents appreciated multiple opportunities to overcome preconceptions that such photography is morbid or otherwise wrong, reporting gratitude that they were asked again and offered more information. In three cases, parents endorsed that professional photographers be called despite parents’ initial negative feelings.

These data suggest that the directive in the literature not to “steer” parental choice [(Henley and Schott 2008), p. 327] ignores the complexity providers face with parents enduring an exceedingly traumatic experience. As Badenhorst and Hughes (Badenhorst and Hughes 2007) note: “At a time when parents are highly aroused and fearful, clinicians should note that parents may struggle to take in information and that it may need to be repeated” [p. 253]. They continue:

The principle of patient autonomy is paramount, as in any medical decision-making, but arguably weakened by the reduced capacity of a parent to make decisions at a time when they are intensely shocked and distressed. Inevitably, in many cases the decision is likely to be heavily influenced by the attending staff [(Badenhorst and Hughes 2007), p. 254].

Recent evidence on the impact of professional demeanor on parental feelings about choosing to hold a stillborn baby likewise indicates the broad scope of provider influence (Erlandsson et al. 2013). In fact, providers are inherently steering patients in many aspects during health care processes; the relevant question is how to best provide evidence-based yet individually respectful care that will ultimately benefit vulnerable, grieving parents.

The answer defies simple formulas that either “overgeneralize” or “hyper-standardize” [(Cacciatore 2011), p. 212], and clearly requires appropriate provider education as well as sensitivity, compassion, and flexibility (Limbo and Kobler 2010). Multiple authors have discussed the importance of perceived support and compassion from professionals [e.g., (Kelley and Trinidad 2012; Hughes and Goodall 2013; Limbo and Kobler 2010)]. “Through a caring relationship with the clinician, a woman can make decisions based on her authentic desires rather than based on fear….Psychoeducation that is conveyed with warmth and honesty about…options such as holding the baby, photographs, or mementos, may give them a sense of informed control” [(Cacciatore 2012), p. 695].

Many parents in the present study noticed and appreciated the sensitive support they received during the crisis of their baby’s death. The present data likewise indicate that parents are sensitive to maltreatment and disappointments. Mindless, coercive, or rigidly protocolized behavior by providers can “intensify parental grief” [(Lang et al. 2011), p. 185; (Kelley and Trinidad 2012; Einaudi et al. 2010; Henley and Schott 2008; Kavanaugh and Moro 2006)]. As Gold (Gold 2007) reported, parents are “acutely aware of how the nurses treated their babies” [p. 233]. Thus providers must offer compassionate and ethical support without imposing personal biases.

When a baby dies, there are many profoundly intense moments which will affect both parents and professionals. Parents’ individual wishes for memorial photography may vary widely, placing significant demands for reflexivity and mindfulness on providers. Inevitably, “the quality of the caregiver-parent relationship is more important than the application of a protocol” [(Einaudi et al. 2010), p. 147]. Clinical wisdom and knowledge, specifically around bereavement care and cultural sensitivity, is crucial to grieving parents (Lang et al. 2011; Cacciatore 2011; Kavanaugh and Moro 2006; Engler et al. 2004; Mander 2009; Roehrs et al. 2008).

### Study limitations

There may be a bias toward photography on the part of parents who have engaged with bereavement support organizations. This study utilized convenience and snowball sampling techniques from lists generated by such organizations. Respondents were overwhelmingly female and predominantly white. For the above reasons, generalizability to all parents grieving a perinatal death cannot be assumed. However, data do accord with previous findings in extant literature.

### Future directions

Research on the male experience in perinatal death is needed (Liisa et al. 2011). Multicultural research is also rare, though the perinatal death rate for African Americans is double the combined mean of other ethnic groups (Kavanaugh and Hershberger 2005; MacDorman et al. 2012). Additionally, a large-scale national study gathered from a variety of sources comparing the experiences of parents in hospitals with and without specific support for bereavement photography may provide compelling data to inform best practice.

## Conclusions

The present study supports and expands findings in extant literature on the importance of perinatal memorial photography for the majority of research participant parents in the United States, adding significant data regarding parents’ preferences, issues of consent, and provider guidance. Providers inherently influence parents during crises. Thus, they should assist grieving parents by gently but clearly imparting the importance of photography with sensitivity for cultural or individual variance in the desire for such mementos. Parents in this study particularly appreciated *assertive* efforts by professionals to encourage and enable photography, including staff approaching more than once for consent or seeking consent from a spouse. However consent is clearly an ethical mandate; its absence can harm.

Parents facing perinatal death should be offered quality, compassionate bereavement photography, optimally by well-trained, professional photographers. Parents should be cognitively and emotionally prepared at the time they receive their pictures; if necessary pictures should be stored safely until parents are prepared and able to request them. Pictures tend to evoke gratitude later—though parents may question this at the time. Parents should also be offered support for their own photo-taking, and nurses should be prepared to assist parents in creating memories. Regardless of the photographer, for the majority of parents losing a newborn, any photos taken sensitively are better than no photos.

This study is in accord with previous research which indicates that quality bereavement photography and the creation of mementos is an “invaluable step in the grieving process” for a majority of parents in U.S. settings [(Williams et al. 2008), p. 338]. Hospitals should endeavor to provide this psychosocial support in the most compassionate and sensitive manner, with flexible, individualized bereavement protocol enacted by educated, mindful professionals intent on patient-centered care. After all, as one bereaved parent said, “Those photographs will be all that is left when the professionals vanish”.

## Authors’ information

CB is a behavioral health clinician in an inpatient psychiatric setting. Her professional interests include psychological trauma, dissociation, and parental grief. She conducted the present research while a graduate student at Arizona State University. JC is an Associate Professor in the School of Social Work at Arizona State University. She researches traumatic death, most often the deaths of babies and children.

## Electronic supplementary material

Additional file 1: **Copy of administered survey.** (PDF 65 KB)
