# Supplementary material for: Best practice in bereavement photography after perinatal death: qualitative analysis with 104 parents
Source: BMC Psychol. 2014 Jun 23;2(1):15. doi: 10.1186/2050-7283-2-15 (PMC4362629; doi:10.1186/2050-7283-2-15)
Supplement: Supplementary file 1 — Additional file 1: Copy of administered survey. (PDF 65 KB) [file 40359_2014_40_MOESM1_ESM.pdf]

All respondents routed to welcome message and the first 5 questions.

Dear Parents:

Hello and thank you sincerely for your interest. We understand that there is no measuring the magnitude of pain and loss after the death of a child. We know that you come to this survey with your own very unique experiences, memories, and feelings. Each person coming to this survey is at a different place in the difficult journey of traumatic grief. We sincerely appreciate your taking time to add your unique voice to our research on the experiences of bereaved parents, and we hope that with this research we will move the world a little closer to a compassionate care model that offers the best support possible to individuals and families in their time of deepest crisis.

I am a graduate student under the direction of Dr. Joanne Cacciatore in the School of Social Work at Arizona State University. The purpose of this study is to better understand a specific aspect of the grief process and support needs of bereaved parents, on the subject of bereavement photography, so as to help improve bereavement care and understanding on the part of human service workers and ultimately the general public.

If you have experienced the loss of your child or children I respectfully invite you to participate in this online survey about your feelings regarding photography after the death of your child or children. You are invited to participate whether or not you had experiences with bereavement photography. Completion of the survey, 11 questions plus demographics, is expected to take an

average of 15-20 minutes. You may choose to make longer or shorter answers. Please know that your participation is greatly appreciated.

Your participation in this study is voluntary. If you choose not to participate or to withdraw from the study at any time, there will be no penalty. You can skip questions if you wish. We ask that you please be 18 or older to participate. Your responses will be anonymous. The results of this study may be used in reports, presentations, or publications but your name will not be known.

Your participation in this research project will provide an opportunity for you to openly express your feelings regarding the death of your child or other loved one. It may help to educate and prepare others for loss experiences, or understand their own grief. This data may add to the body of scientific knowledge about providing competent interventions to those experiencing traumatic bereavement. There are no foreseeable risks to your participation, but remembering and describing painful experiences can certainly bring up feelings of sadness, grief, and longing. We urge you to utilize the resources provided to connect with other bereaved parents and bereavement specialists, and to take time to give yourself excellent self-care.

If you have any questions concerning the research study, please feel free to contact the principal investigator and research supervisor Dr. Joanne Cacciatore at (XXX) XXX-XXXX, or me, Cybele Blood, at (XXX) XXX-XXXX. [DELETED FOR PRIVACY IN PUBLICATION]. If you have any questions about your rights as a subject/participant in this research, or if you feel you have been placed at risk, you can contact the Chair of the Human Subjects Institutional Review Board, through the ASU Office of Research Integrity and Assurance, at (480) 965-6788.

Return of the questionnaire will be considered your consent to participate in the Parental Bereavement Photography Study.

If you have lost more than one child, we ask that you please fill out a survey for each child. There is also room in the short essay answer boxes for you to discuss your experiences overall. Thank you.

1. How old was your child at the time of his or her death? (Can include minutes, hours, days, years, or died before or during birth)

2. In what year did you lose your child?

3. What was the cause of your child's death, if known? (Can put "unknown" or add explanation)

4. After or at the time of the death of your child, did anyone (professional, family, or friend) ask if you wanted to take photographs, or have photographs taken, of your child?

5. Did you (or your family) take photographs, or request or allow others to take photographs of your child, or were any photographs taken for any reason during or after the time of your child's death?

___

> **If No: (Otherwise survey automatically routes to #8)**
>
> **6. Some parents report changing their mind later and wishing they had photographs. Are you content with your decision not to take photographs or have photographs taken of your child?**

If No: Could you describe a bit about why you wish you had made a different choice?

7. Is there anything else about your experience with not taking or not having photographs of your child during or after his or her death that you would like the researchers, readers, or health service professionals to know?

If Yes: Please take as much space as you would like

___

8. Did you receive and/or keep these photos, and if so, how many photos do you have? (Approximate # is fine)

9. Can you list all the ways you have used this photograph or these photographs, or the ways others have used the photographs (for examples: to look at and think about him or her, to show others, to put in a shrine, used for police investigation, to post on a website for bereaved parents, to put in a memorial service, etc…)

10. Could you please say a bit about what this photograph means or has meant to you, or what these photographs mean or have meant to you?

11. Have you gotten feedback from anyone about your decision to take photographs or allow photography of your child after his or her death, or feedback about your later use of the photograph(s) of your child?

If Yes: Could you describe a bit about the feedback you have gotten?

12. Were you present during the taking of the photo(s)? and

Would you please describe a bit about what that (being there/not being there) was like for you and/or what it meant to you?

13. Is there anything else about your experience with photographs of your child during or after his or her death that you would like the researchers, readers, or health service professionals to know?

If Yes: Please take as much space as you would like

All respondents routed to the following questions:

14. Please provide us with some additional data for statistical purposes

What is your age?

What is your sex (or gender)

What is your religion and/or spirituality as you identify it?

What is your race/ethnicity?

Do you live in a city, in a small town, or in a rural area?

Do you have other children? Will you please share how many and their ages?

All respondents routed to closure message of thank you for participation
